# Synthesis of biogenic silver nanoparticles from apricot kernel extracts for the colorimetric determination of gold

**DOI:** 10.55730/1300-0527.3768

**Published:** 2025-08-18

**Authors:** Ayça GİRGİN, Hilal AKBIYIK, Buse Tuğba ZAMAN, Omid NEJATI, Ayça BAL ÖZTÜRK, Gülten ÇETİN, Sezgin BAKIRDERE

**Affiliations:** 1 Department of Chemistry, Faculty of Arts and Science, Yıldız Technical University, İstanbul, Turkiye; 2 Neutec Pharmaceutical, Yıldız Technical University Teknopark, İstanbul, Turkiye; 3 Institute of Health Sciences, Department of Stem Cell and Tissue Engineering, Institute of Health Sciences, İstinye University, İstanbul, Turkiye; 4 Stem Cell and Tissue Engineering Application and Research Center (ISUKOK), İstinye University, İstanbul, Turkiye; 5 CanChip Research and Development Laboratory, Potsdam, Bradenburg, Germany; 6 Department of Analytical Chemistry, Faculty of Pharmacy, İstinye University, İstanbul, Turkiye; 7 Turkish Academy of Sciences (TÜBA), Ankara, Turkiye

**Keywords:** Gold, biogenic silver nanoparticles, UV-Vis spectrophotometer, colorimetric sensor, spectrophotometry

## Abstract

Gold is abundant in nature, however, precise and reliable analytical methods for its detection are required stemming from its increasing prevalence in environmental, biological, and industrial systems, as well as the growing interest in understanding its function in living organisms and its effects on human health. This study investigates the use of biogenically synthesized silver nanoparticles (AgNPs) for the preconcentration and determination of gold ions prior to determination by UV-Vis spectrophotometry. AgNPs were synthesized by reducing silver nitrate through the use of an apricot kernel extract as both reducer and stabilizer agent. The colloidal yellowish AgNPs interacted with gold ions, leading to a distinct color change and a considerable decrease in the surface plasmon resonance (SPR) intensity at 415 nm in the UV-Vis absorption band, indicating a highly sensitive and selective colorimetric detection of gold ions. Under optimized conditions, the proposed method achieved satisfactory limit of detection (LOD) and limit of quantification (LOQ) values of 2.9 and 9.7 mg/L, respectively. A matrix matching calibration strategy was used to enhance quantification accuracy, resulting in satisfactory percent recoveries from waste mud samples (88–112%). Overall, the results validated the developed method as a green, simple, rapid, and accurate analytical approach to the determination of gold.

## 1. Introduction

Gold (Au) is a highly valuable noble metal with unique features that has been greatly utilized for the making of fine jewelry, decorative objects, and accessories for thousands of years [1]. Furthermore, its excellent conductivity and high resistance to corrosion make it a very useful material for electronic applications [2]. Among the natural heavy metals, gold exhibits excellent photothermal, catalytic and biocompatible properties that have been exploited in the fields of medicine, chemistry, and biology [3]. In medicine, gold plays a key role in the treatment of severe diseases such as cancer, arthritis, tuberculosis, malaria, human immunodeficiency virus (HIV), and asthma, due to its anti-inflammatory properties [4]. In addition, gold nanoparticles have been employed in numerous other applications, including drug/gene delivery systems, cosmetic products, biomolecular screening, and biosensors [5]. In its oxidized state (Au (III)), gold binds strongly to enzymes, amino-acids, proteins, and DNA due to its high thiophilicity, with an adverse impact on their biological activity [6]. In addition, Au (III) ions are considered a pollutant due their inhibition of enzymatic activity and their prevention of DNA fragment separation in both plants and animals [7]. Furthermore, soluble gold salts (e.g. gold chloride) can cause significant damage to the kidneys, liver, spleen, and lungs of living organisms [8]. In light of these negative effects on the environment and human health, as well as the accompanying economic constraints, there is a clear need for simple and practical approaches to the detection and removal of gold.

Several traditional techniques are available for the detection of gold, and while they produce highly sensitive and accurate results, the sample pretreatment procedures are complex and time consuming [9].

With the advances in nanotechnologies, metallic nanoparticle-based colorimetric sensors have gained popularity for the detection of a wide variety of analytes, including biomolecules, drugs, metal ions, and pesticides [10]. Due to their easy operation, low cost, simplicity, naked eye detection, and rapidity, colorimetric sensors have come to be widely employed over the past few decades [11]. This method takes advantage of an optical phenomenon known as Surface Plasmon Resonance (SPR) for the detection of metal nanoparticles and their aggregation activity [12]. As the color of solution changes due to a significant alteration in its optical properties resulting from aggregation processes, the shift in the SPR absorption band can be easily monitored with an appropriate spectrophotometer [12–14]. Silver nanoparticles (AgNPs) are usually preferred over other metallic nanoparticles as a visual output detector for the quantitative determination of different target analytes due to their unique optical properties and high extinction coefficients in the visible region [13–15]. They are regularly utilized in food industries, water treatment systems, pomades, gels, healthcare fabrics, cotton buds, and chemotherapeutics due to their excellent antifungal and antimicrobial efficacies [16].

AgNPs are synthesized through physical and chemical procedures, including ultrasound irradiation, chemical vapor deposition, explosion, deposition-precipitation, laser ablation, sol-gel, coprecipitation, evaporative cooling, impregnation, and chemical reduction [17]. These traditional techniques, however, require the use of poisonous compounds and extreme environments, resulting in ecological challenges and toxic waste. Green chemistry synthesis approaches are more environmentally friendly, utilizing the natural extracts found in polyphenolic substances, however synthesis can be difficult due to the variations in the compounds sourced from different sources [18]. For this reason, many researchers have turned to biological synthesis methods that are non-toxic, eco-friendly, simple, and cost-effective [19]. Various sources can be utilized for the synthesis of AgNPs, including algae, essential oils, yeast, microorganisms, plants, biomolecules, and mushroom extracts [20]. Among these substances, plant extracts have been highly acclaimed as biogenic precursors for the synthesis of AgNPs due to the presence of such varied chemical components as alkaloids, flavonoids, phenolic compounds, and sterols [21], allowing them to play both reducer and stabilizer roles [22]. In recent years, the use of plant species for the production of nanoparticles has emerged as the most reliable environmentally sustainable strategy [23]. The stability, morphology, and size of the produced nanoparticles can be accurately controlled by adjusting the temperature, pH, plant extract concentration, metal salt solution, and incubation period during synthesis [24]. Previous studies have employed different parts of plants, including seeds, leaves, stems, flowers, and roots, as synthesizing reagents in the preparation of metallic nanoparticles [25]. The present study analyzes the apricot kernel, the shell of which contains lignin, cellulose, hemicellulose, and small quantities of other organic compounds [26]. This makes it highly suitable for the reduction of metal ions and the production of metal nanoparticles.

Briefly, apricot kernel extract was used as a biogenic reducing and stabilizing agent for the synthesis of AgNPs, which was then used for the colorimetric detection and preconcentration of gold ions. The optimum parameters of the study were determined through univariate optimization experiments, and were applied for the sensitive determination of gold by UV-Vis spectrophotometry. The accuracy of the proposed method was demonstrated through spiked recovery experiments on waste mud and soil samples.

## 2. Experimental parameters

Experimental parameters including AgNP concentrations, mixing type and duration, and pH of the buffer solution were optimized to enhance the sensitivity and selectivity of the SPR-based colorimetric assay for the detection of gold ions. The effect of each parameter was evaluated based on the single variant approach with a minimum of three measurements, and blank corrections were performed for all measurements to enhance the accuracy of the experimental results. The optimization studies made use of a standard 25 mg/L Au aqueous solution. The highest surface plasmon resonance absorption peak intensity value with a favorably low percent relative standard deviation (%RSD) was selected as the optimum parameter for each optimization step.

### 2.1. Instrumentation

All solutions were placed in 1.0 cm optical path quartz cuvette, and their UV-Vis absorption spectra were obtained using a Shimadzu UV-2600 model UV-Vis spectrophotometer at room temperature. To achieve the maximum interaction between the AgNPs and Au ions in the solution, a Kerman Lab 51 model orbital and reciprocating mechanical shaker was employed for the mixing procedures. For the preparation of the biogenic extract, the shells of apricot kernels were dried in a Heraeus D-6450 brand drying oven (Heraeus, Germany) for 24 h, and a Fakir Aromatic model mixer grinder (Germany) was then used to grind the dried apricot kernel shells. An IKA C-MAG HS7 (Staufen, Germany) model magnetic stirrer with a hot plate was used throughout the AgNPs synthesis procedure.

### 2.2. Chemicals and reagents

A stock solution of Au (1000 mg/L) in 2.0% (w/v) HCl was purchased from High Purity Standards (South Carolina, USA) following the recommendations in ISO/IEC 17025 Guide 34, and diluted with water obtained from a Milli-Q® Reference Ultrapure Water Purification System (USA). to prepare the calibration and working standard solutions. The biogenic synthesis of AgNPs was performed using silver nitrate purchased from Merck (Germany), where the reduction of Ag^+^ to Ag° metallic state and subsequent stabilization was achieved using an aqueous extract apricot kernel. The buffer solutions tested in the study were prepared from potassium hydrogen phthalate (0.40 M), tris-hydroxymethyl amino methane (0.50 M), and sodium tetraborate (0.060 M) solutions, and adjusted to the final pH values with dilute hydrochloric acid and sodium hydroxide solutions. All salts were purchased from Merck (Germany).

### 2.3. Silver nanoparticle synthesis and characterization

#### 2.3.1. Preparation and collection of plant extract

The biogenic synthesis method was applied for the preparation of AgNPs, for which the raw apricot kernels were first thoroughly rinsed with deionized water to eliminate any dust and surface impurities derived from the raw apricots. The washed apricot kernels were peeled, and the brown-colored surface skin was dried in an oven for 24 h at 50–55 °C. The dried apricot kernel skins were then ground into fine powder in a mixer grinder. Subsequently, about 8.0 g was taken from the powdered sample into a 100 mL beaker, mixed with 50 mL deionized water, and heated for 1.0 h at 70 °C. The resulting solution was allowed to cool at room temperature and then filtered to remove residual particles from the aqueous extract. Finally, the solution was centrifuged for 10 min at 3000 rpm for the collection of a clear yellow colored aqueous extract that was then stored at +4.0 °C. The extract was used as both reducing and stabilizing agent for the synthesis of AgNPs.

#### 2.3.2. Synthesis of silver nanoparticles with apricot kernel extract

Based on the modified procedure reported in the literature for the biogenic synthesis of AgNPs [27], 10 mL of freshly prepared 1.0 mM AgNO_3_ solution was taken into a beaker and boiled to a temperature of 70 °C. Then, 2.0 mL of the prepared extract was added to the AgNO_3_ solution drop by drop with vigorous stirring at 70 °C, and the mixture was kept in a magnetic stirrer for 2.0 h. The initial colorless mixture turned brownish yellow in approximately 90 min, indicating the formation of AgNPs through the reduction of Ag^+^ ions by the extract of apricot kernel. The obtained colloidal AgNP solution was then kept in dark conditions at room temperature until its use in method optimization and validation experiments. The resulting AgNPs were characterized through Fourier Transform Infrared Spectroscopy (FTIR), Transmission Electron Microscopy (TEM), Dynamic Light Scattering (DLS), and Zeta Potential analysis. The obtained results confirmed the synthesis of AgNPs based on a comparison with previous literature reports, as stated in the applied procedure [28].

#### 2.3.3. Waste-mud sample preparation

Waste mud samples were taken from a jewelry production company (İstanbul, Türkiye), while soil samples were collected from two different locations within the Yıldız Technical University Davutpasa Campus (İstanbul, Türkiye). These samples were used to validate the ability of the developed method to accurately quantify analytes in complex sample matrices. The samples were dried thoroughly in an oven at 55–60 °C for 24 hours and ground in a mixer grinder to obtain fine particles. Then, 1.0 g of waste mud and 10 g soil samples were mixed, considering their abundance in nature. The waste mud and soil samples were blended using a clean spatula until a homogeneous mixture was obtained. After making sure that the waste mud sample was homogeneously distributed within the soil sample, the samples were placed in an oven to remove the moisture. Afterwards, 1.0 g of the homogeneous mixture was weighed into a clean test tube and completed to 20.0 g with deionized water. To ensure homogeneity and to optimize the transfer of gold ions into the solution, the sample was sonicated for 10 min and then placed in a mechanical shaker for 15 min. The final solution was filtered using filter paper (125 μm pore size) and the filtrate was completed to a final volume of 25 mL with deionized water. The extraction process was determined, and the sample filtrate was diluted five times. The sample extract was then spiked with different concentrations for the recovery experiments.

#### 2.3.4. Colorimetric assay for gold

A simple procedure was used to prepare standard sample solutions for colorimetric measurement. First, 0.75 mL of the colloidal AgNP solution (0.50 mM) was pipetted into a clean 15 mL centrifuge tube followed by the addition of 100 μL of aqueous standard/sample solution. The resulting solution was agitated on a mechanical shaker for 30 s to obtain a homogenous mixture. Finally, 0.70 mL of the prepared homogenous solution was pipetted into a quartz cuvette and the absorption intensity was monitored using a UV-Vis spectrophotometer at 415 nm wavelength.

## 3. Results and discussion

### 3.1. Sensing ability of AgNPs to Au

It is widely recognized that biogenically synthesized AgNPs have powerful UV-Vis absorption spectra and exhibit characteristic color changes in aqueous solutions as a result of their surface plasmon resonance (SPR) [29]. In previous studies, Firdaus et al. [30] reported that the sensing mechanism between silver nanoparticles and the target analyte can be described by the following reaction:


$$2\text{Ag}+2{\text{Me}}^{2+}\;\;\;2{\text{Ag}}^{+}+{{\text{Me}}_{2}}^{2+}$$

The SPR sensing mechanism of AgNPs can be explained by oxidation, aggregation, and size/morphological property change mechanisms [31]. The standard reduction potentials of Au^3+^/ Au^+^, Au^+^/Au, and Ag^+^/Ag pairs at 25°C are +1.40 E^q^/V, +1.69 E^q^/V, and +0.80 E^q^/V, respectively, which suggests that the reduction-oxidation reaction can occur spontaneously [32]. This finding suggests that the SPR mechanism is based on oxidation, and confirms the interaction between Au ions and the AgNPs.

In light of this information, the possible redox reaction might be presented in two ways [32]:


$$\begin{array}{l} \text{Ag}+{\text{Au}}^{+}\to {\text{Ag}}^{+}+\text{Au}\\ 3\text{Ag}+{\text{Au}}^{3+}\to 3{\text{Ag}}^{+}+\text{Au} \end{array}$$

**Figure 1:**
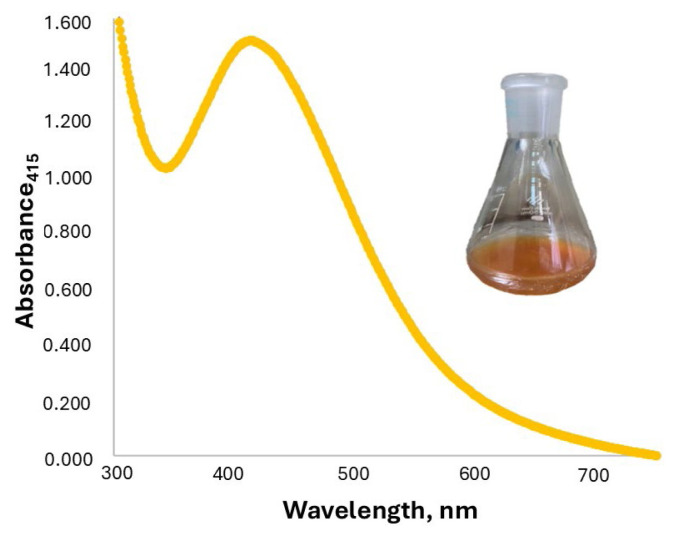
Spectrum of the SPR absorption band of the synthesized AgNPs.

The absorption spectrum of the colloidal suspension of AgNPs exhibits a surface plasmon resonance absorption band within the scanning range of 250–750 nm. This optical property causes the AgNPs to change color from transparent to brownish yellow in the visible region. As shown in Figure 1, the most intense SPR absorption peak of 0.50 mM AgNPs was observed at 415 nm, along with the characteristic color, indicating the successful synthesis of AgNPs.

Owing to its broad-spectrum sensing capabilities and applications, an AgNPs-based colorimetric sensor was utilized for the quantification of gold ions. When the AgNP solution was introduced to varying concentrations of gold ions, a gradual decrease was noted in the intensity of the SPR band at 415 nm, which was attributed to the redox reaction mechanism between AgNPs and the target analyte. Notably, as the concentration of gold ions increased, the intensity of the SPR band at 415 nm gradually decreased. Moreover, visual color changes were evident in the solution mixture of AgNPs and Au ions and reached a colorless state with the addition of low concentrations of the gold standard solution. This can be explained by the color turning from yellow to white as the Ag ratio increases in the Ag-Au alloy. A color transition from colorless to yellow was observed with increasing Au concentrations in the solution containing AgNPs and Au ions [33].

### 3.2. Concentration of AgNPs

**Figure 2:**
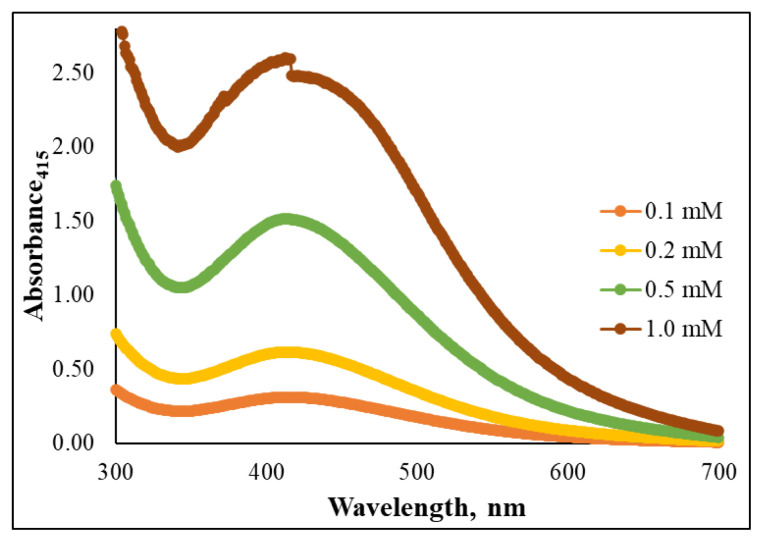
Effect of AgNP concentration on the SPR band peak shape and absorbance value.

AgNP concentration is a highly critical parameter that can influence the efficiency of the developed system due to its effect on the SPR band peak shape and absorbance value, depending on the particle size and morphology of the AgNPs. For this reason, the effect of AgNP concentration on the output of the method was evaluated by testing 1.0, 0.50, 0.20, and 0.10 mM concentrations. The recorded results revealed a gradual decrease in SPR absorption peak intensity at lower AgNP concentrations, while higher AgNO_3_ levels produced more AgNPs, leading to higher absorption efficiency. Maximum absorption was achieved at 415 nm with the 1.0 mM AgNP concentration, although peak distortion was observed as displayed in Figure 2. The 0.50 mM AgNP concentration yielded a narrow SPR absorption peak with high intensity in the visible region. Accordingly, 0.50 mM AgNP was selected as the optimum concentration due to its superior sensitivity for the proposed AgNP based colorimetric sensor.

### 3.3. Buffer type

**Figure 3:**
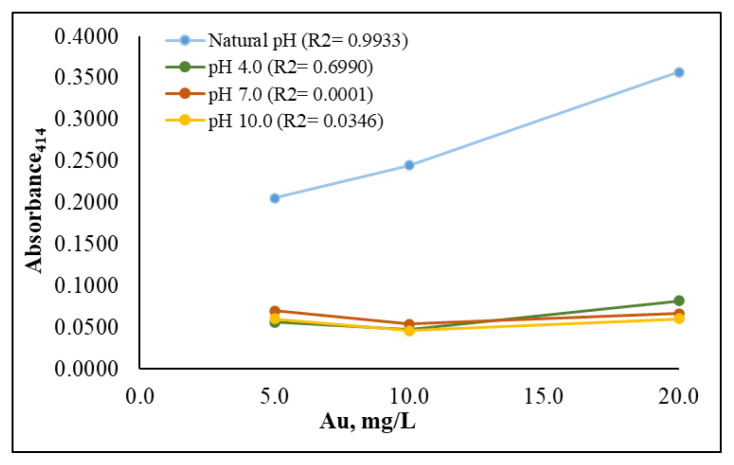
Effect of the use of a buffer solution on the determination of gold ions.

The pH of an aqueous solution has the potential to alter the surface charge of AgNPs, and consequently, to impact their aggregation, dissolution, and overall stability in the solution [34]. It is therefore important to optimize the solution’s pH to ensure nanoparticle stability and to prevent aggregation during the analytical procedure. The role of buffer solutions in providing stability and resistance to pH change was evaluated through the addition of pH 4.0, 7.0, and 10 buffer solutions, representing the acidic, neutral, and basic regions of the pH scale, respectively. Each pH buffer level was tested at three linearly increasing concentrations (5.0, 10, and 20 mg/L) to evaluate the linearity of their plots, and the results were compared with that of a non-buffered solution, herein referred to the natural medium. As shown in Figure 3, the absorbance values recorded for the buffered solutions did not increase proportionally with concentration, resulting in poor linearity. The lack of linearity observed with the pH 7.0 and pH 10.0 buffers may be attributed to the salts used in the preparation of these the buffer solutions. For example, borax salt is utilized in the preparation of a pH 10.0 buffer, but is also employed in the synthesis of silver borate nanoparticles, and the interaction between AgNPs in the medium and borate ions may have prevented AgNPs from responding to the gold ions [35]. Similarly, tris( hydroxymethyl) aminomethane, a component of the pH 7.0 buffer system, can form complexes with silver [36]. Furthermore, this salt has been used to determine the presence of gold ions in water [37]. The interactions of borax salt with both silver and gold provides a plausible explanation for the failure to achieve linearity at pH 7.0. The natural medium, unlike the buffered solutions, exhibited absorbance values that correlated to the tested concentration, achieving high linearity. Further experiments were performed without the addition of buffer solutions, which cut down on the consumption of extra chemicals in the procedure.

### 3.4. Mixing method and duration

**Figure 4:**
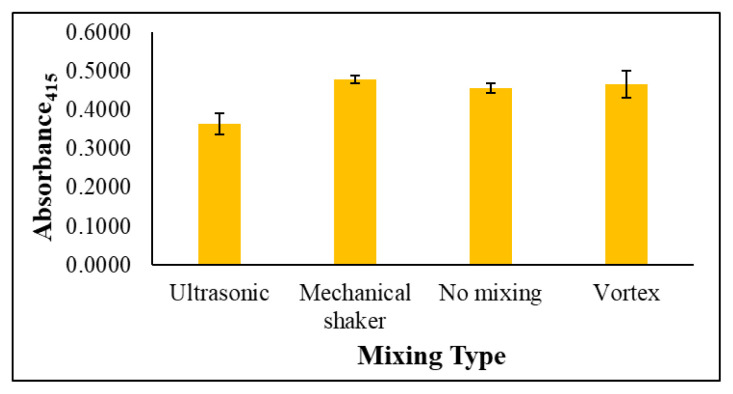
Effect of different mixing types on the determination of gold ions (25 mg/L), n = 3 for error bars.

To ensure the effective and homogenous distribution of metal ions throughout the AgNP solution for maximum interaction, different mixing methods were tested to identify the one that produced the best responses. To this end, the solutions were mixed using different mixing tools for 1 min prior to measurement. The tested mixing methods were ultrasonication, mechanical shaking, and vortexing, and the absorbance values recorded for each were compared with an unmixed sample, as shown in Figure 4. The mean absorbance value achieved through the ultrasonic method was lower than that of the other mixing types. No notable difference was recorded between the results obtained through vortexing, mechanical shaking, and no mixing, although the mechanical shaker recorded the lowest standard error, as can be seen from the error bars in Figure 4, and so this method was selected for further experiments.

**Figure 5:**
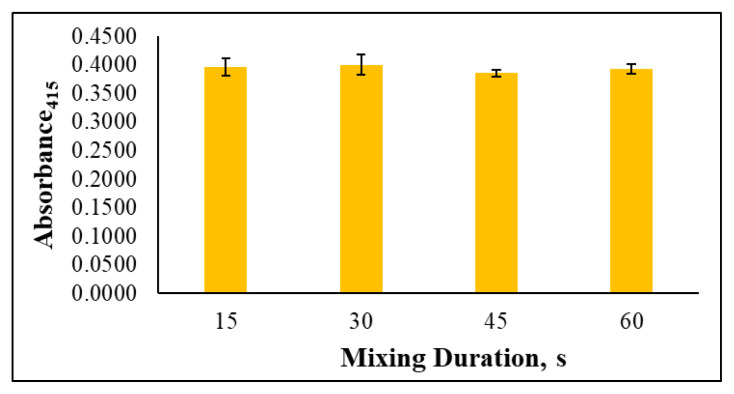
Effect of mixing duration on the determination of gold ions (25 mg/L), n = 3 for error bars.

The mechanical shaking method was then tested at different mixing durations to determine the optimum one that maximizes the interaction between the analyte in solution and the AgNP solution. Mixing durations of 15, 30, 45, and 60 s were tested using equivalent standard solutions. The recorded absorbance values plotted in Figure 5 reveal no notable differences in the results obtained with different mixing durations. To ensure homogeneity in the procedure, a 30 s mixing duration was selected as the optimum and final parameter of the optimization experiments.

### 3.5. Evaluation of the analytical performance of the developed method

The optimized experimental parameters for the quantitative detection of Au ions and sensitivity of the generated colorimetric assay are summarized in Table 1.

Calibration standard solutions of Au (observed as yellow-colored colloidal suspension with AgNPs) were analyzed to record their SPR absorption peaks at 415 nm using a UV-Vis spectrophotometer. A gradual color change was noted from yellow to transparent. The calibration plot developed for the analyzed Au standard solutions was used to validate the optimized method. The validation parameters, including limit of detection (LOD), limit of quantification (LOQ), precision (%RSD), linear dynamic range (LDR), and coefficient of determination (R^2^), were evaluated under the optimized conditions. The mathematical formulas used for the calculation of LOQ and LOD values were $\frac{\sigma }{m}\times 10$ and $\frac{\sigma }{m}\times 3$, respectively, in which σ is the standard deviation of the blank measurements, and m represents the slope of the calibration plot. The SPR absorption peak at 415 nm was evaluated against the Au-AgNP solution concentrations in the 10–100 mg/L linear range, and it was apparent from the results that gold can be quantified using this method over a wide linear range. This calibration plot has a correlation coefficient (R^2^) of 0.9963, equation y = −0.0026x + 0.3868, and %RSD value in the range of 0.10–0.85%. The LOD and LOQ values of the proposed method were calculated as 2.9 and 9.7 mg/L, respectively. The Blue Applicability Grade Index (BAGI) [38] was evaluated based on a comparison with the BAGI values reported by other gold determination studies in the literature presented in Table 2 [6, 39–41].

### 3.6. Recovery

The method developed for the colorimetric detection of Au ions was subjected to spike recovery experiments to evaluate its validity and feasibility for use in real sample analysis operations. The spiking experiments were performed using a waste-mud sample, as described in the experimental section. When analyzing real samples, the response of target analytes may be supressed by potential interferences, particularly when working with environmental samples that have complex matrices. It is very important, therefore, to use quantification strategies that eliminate/reduce interferences from sample matrices. The sample extract was spiked at four different concentrations, and the percent recoveries calculated using an external calibration method (standard solutions prepared with deionized water) were highly irregular, indicating a strong matrix effect on the quantification of the analyte. In an attempt to mitigate the matrix effect, a matrix-matching calibration approach was adopted to quantify the sample extract spiked at four concentrations between 10 and 100 mg/kg. In this approach, a different waste-mud fused soil sample extract was used for the preparation of calibration standards, which were plotted and achieved a satisfactory linearity. The concentrations of the spiked soil samples were then calculated using the linear equation of the matrix-matched calibration, and the relative recoveries were subsequently determined. The matrix-matching approach led to improved percent recoveries for the spiked samples (88–112%), as detailed in Table 3.

## Conclusion

The economic value of Au and the potential effects of this element on both the environment and human health, even at trace levels, makes it prudent to maximize the sensitivity and accuracy of analytical detection methods. This study presents a simple and direct colorimetric detection approach based on a combination of aqueous Au solutions with biogenic AgNPs, synthesized using apricot kernel extracts. The parameters of the method were optimized to enhance the absorption intensity of Au, and applying the optimum conditions to aqueous standards yielded detection and quantification limit values of 2.9 and 9.7 mg/L, respectively. The optimized colorimetric method was also applied to waste-mud sample extracts in spike recovery experiments. The percent recoveries calculated for the spiked extracts using the external calibration method did not fall within acceptable limits, which was attributed to interference effects; however, the matrix-matching method was used to correct this effect to attain recoveries in the 88–112% range. On the ecological side, the greenness of the developed method was found to be environmentally friendly based on its score of 0.7 using the Analytical GREEnness (AGREE) calculator … in accordance with the 12 principles of green analytical chemistry. The proposed method can be considered a rapid, cost-effective, ecologically friendly, real-time, and easy-to-use approach to the determination of gold using AgNPs.

## Figures and Tables

**Table 1:** Optimum parameter values for the developed method.

Parameters	Values
AgNP concentration	0.50 mM
pH of buffer solution	pH 6.5 for natural pH of the colloidal AgNP solution
Mixing type, and duration	Mechanical shaker, 30 s

**Table 2:** Comparison with BAGI values reported in previous gold determination studies.

Blue Applicability Grade Index (BAGI)	This study	[39]	[40]	[6]	[41]
**Type of analysis**	Quantitative and confirmatory	Quantitative and confirmatory	Quantitative and confirmatory	Quantitative and confirmatory	Quantitative and confirmatory
**Multi- or single-element analysis**	Single Element	Single Element	Single Element	Single Element	Single Element
**Analytical technique**	UV–vis spectrophotometry	Inductively coupled plasma mass spectrometry	Inductively coupled plasma optical emission spectrometry	UV–vis spectrophotometry or Samsung A50 phone	Flame atomic absorption spectrometry
**Simultaneous sample preparation**	1	1	1	1	1
**Sample preparation**	Not require or on-site sample preparation if required	Ion imprinted polymer magnetic solid phase extraction	Solid phase extraction (Column SPE, micro coloumn)	Not require or on-site sample preparation if required	Ion pair-dispersive liquid–liquid microextraction
**Samples per h**	>10	2–4	>10	>10	5–10
**Reagents and materials**	Need to be synthesized in the lab with common instrumentation and in a simple way	Need to be synthesized in the lab with advanced equipment or know-how	Need to be synthesized in the lab with advanced equipment or know-how	Need to be synthesized in the lab with common instrumentation and in a simple way	Commercially available reagents not common in QC labs
**Preconcentration**	No preconcentration require. Required sensitivity and /or legislation criteria are met directly.	Preconcentration required. Required sensitivity is met with one-step preconcentration.	Preconcentration required. Required sensitivity is met with one-step preconcentration.	No preconcentration require. Required sensitivity and /or legislation criteria are met directly.	Preconcentration required. Required sensitivity is met with one-step preconcentration.
**Degree of automation**	Manual treatment and analysis	Semi-automated with common devices	Semi-automated with common devices	Manual treatment and analysis	Manual treatment and analysis

**Table 3:** Percent recoveries of Au spiked to waste-mud fused soil samples, calculated using matrix-matching calibration.

Spiked Au concentration in soil samples, mg/kg	Calculated Au concentration in soil samples, mg/kg	Recovery% ± SD n = 3
24.3	26.1	107.4 ± 1.0
51.5	57.4	111.4 ± 3.1
77.1	81.3	105.4 ± 1.8
102.6	90.4	88.1 ± 0.9

## Data Availability

Data can be provided upon reasonable request.
